# Unlocking the potential of *Metschnikowia pulcherrima*: a dive into the genomic and safety characterization of four plant-associated strains

**DOI:** 10.1007/s00253-025-13515-0

**Published:** 2025-05-29

**Authors:** Ilaria Larini, Massimo Ferrara, Eleonora Troiano, Veronica Gatto, Giuseppina Mulè, Nicola Vitulo, Vittorio Capozzi, Elisa Salvetti, Giovanna E. Felis, Sandra Torriani

**Affiliations:** 1 Department of Biotechnology, University of Verona, Strada Le Grazie 15, Ca’ Vignal 2, 37134 Verona, VR Italy; 2 Institute of Sciences of Food Production (ISPA), National Research Council (CNR), Via Amendola 122/O, 70126 Bari, Italy; 3 Institute of Sciences of Food Production (ISPA), National Research Council (CNR), Via Michele Protano, 71121 Foggia, Italy; 4 Department of Biotechnology, VUCC-DBT, Verona University Culture Collection, University of Verona, Strada Le Grazie 15, Ca’ Vignal 2, 37134 Verona, VR Italy

**Keywords:** Antimycotic resistance, Biocontrol, Genomic analysis, *Metschnikowia pulcherrima*, Safety assessment, Pulcherrimin

## Abstract

**Abstract:**

*Metschnikowia pulcherrima* includes strains of applied agro-food interest, particularly due to the antimicrobial activity against plant pathogens, contribution to the aroma of fermented beverages, and preliminary evidence related to probiotic activity. This biotechnological relevance sheds new light of interest on the biology of this yeast. To better understand and expand its biotechnological potential and applicability, the genomes of *M. pulcherrima* NRRL Y-7111^ T^, NRRL Y-48695, CBS 10357, and NRRL Y-48712 were sequenced, and de-novo assembled. Between 10,671 and 14,548 genes were predicted and the cooperative genomic analyses were integrated with experimental assessments relating to traits relevant for biotechnological application and safety. In silico and in vitro safety assessment revealed intermediate sensitivity for itraconazole; furthermore, variants of the genes related to pulcherrimin production and transport were found in all the genomes. Moreover, an arsenal of carbohydrate-active enzymes (CAZymes) was unravelled, and their predicted localization was investigated. This study expands the body of knowledge on *M. pulcherrima*, including traits relevant for defining its safety as a bioresource, which is a pivotal aspect for its possible inclusion in the European Food Safety Authority (EFSA) Qualified Presumption of Safety (QPS) list and its application in REgulated food/feed PROducts (REPRO) both in the European Union & aligned European countries.

**Graphical Abstract:**

**Figure:**
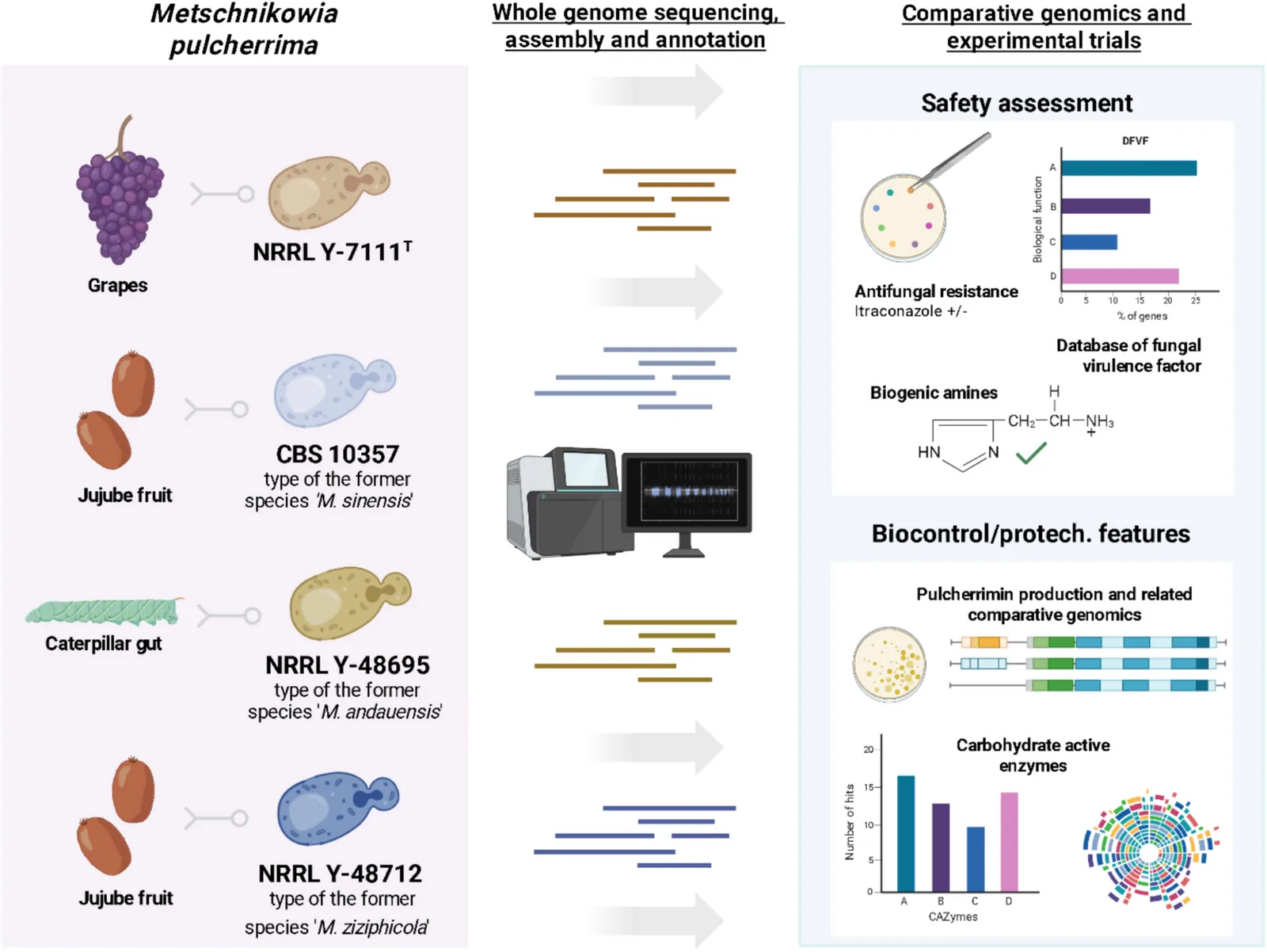

**Key points:**

*• A pipeline for genomic characterisation and safety assessment of unconventional yeasts, using M. pulcherrima as a model species was developed.*

*• M. pulcherrima strains can be considered safe and safety data can be used to develop a body of knowledge to include M. pulcherrima in EFSA QPS list.*

*• Analysis of the predicted localization of CAZymes allowed the detection of compounds as potential biological control agents.*

**Supplementary Information:**

The online version contains supplementary material available at 10.1007/s00253-025-13515-0.

## Introduction

*Metschnikowia pulcherrima* is a versatile, non-conventional yeast isolated from various sources, such as plant–insect interfaces, fruits (e.g., grapes) and orchards (Lachance 2016; Tatay-Núñez et al. 2024; Janisiewicz et al. 2001). Strains of this species have diverse applications in biotechnology, as they produce hydrolytic enzymes, organic acids and aromatic compounds, which improve the aroma and the color of fermented plant-based beverages. Furthermore, they also release a complex of pulcherriminic acid and ferric ions, that confers *M. pulcherrima* antagonistic properties against various human pathogens, fungal species, spoilage yeasts and moulds, making it a promising biocontrol resource (Rahmat et al. 2024; Sipiczki 2020, 2006; Pawlikowska et al. 2019; Pawlikowska and Kręgiel 2017; Türkel and Ener 2009). In addition, a preliminary characterization with respect to the potential for probiosis has been proposed for some strains of this species (Agarbati et al. 2020; 2024). Besides agri-food applications, some *M. pulcherrima* strains can also be suitable for industrial exploitation as they could reach large-scale lipid-yield production from the conversion of low-cost volatile fatty acids (VFAs) in limiting nitrogen environment (Němcová et al. 2021; Abeln and Chuck 2021). Further, thanks to the production of antimicrobial compounds (such as the pulcherriminic acid), they can work in non-sterile conditions, allowing a more sustainable waste treatment, resource recovery, and less pollutant discharge (Li et al. 2021).

From a taxonomic point of view, this species was grouped with other closely related ones in the so-called *M. pulcherrima* clade (Sipiczki 2020, 2022a; Lachance 2016). Early in 2022, all these species, except *Metschnikowia chrysoperlae*, were reclassified as *M. pulcherrima* (Sipiczki 2022b), and this taxonomic change was further supported by Troiano et al. (2023), where a comparative genomic analysis of seven *M. pulcherrima* clade strains revealed the absence of single-copy markers for species differentiation. Moreover, a recent study (Sipiczki et al. 2024) investigating the intragenomic, intergenomic, and phenotypic diversity of 37 M*. pulcherrima* isolates showed that the molecular differences did not clearly correlate with the phenotypes observed and failed in differentiating strains, highlighting that these genomes may be chimeric. Further these strains seem to be prone to reversible segregation from a phenotypic point of view, following a non-standard evolutionary model. Genome sequences are keystone data to explore the applicability of microbial bioresources: they are crucial for unequivocal taxonomic identification and for guiding a comprehensive safety assessment, which is the principal regulatory concern related to microorganisms added deliberately to the food or feed chain, as requested by the European Food Safety Authority (EFSA) (Miguel et al. 2022; Binati et al. 2021; EFSA guidance 2018). Although *M. pulcherrima* is reported in the “inventory of microbial food cultures with safety demonstration in fermented food products’’ released by the International Dairy Federation (IDF) (IDF Bulletin 2022), a deep safety analysis is necessary, as no *Metschnikowia* spp. is currently included in the EFSA Qualified Presumption of Safety (QPS) list (EFSA 2025).

Previous comparative genomic analysis of *M. pulcherrima* has been focused on a few strains (Hershkovitz et al. 2013; Piombo et al. 2018; Wang et al. 2020; Gore-Lloyd et al. 2019; Rahmat et al. 2021, 2024). Since those do not represent the former species of the previous *M. pulcherrima* clade, the aim of this research is to study the genomes of other four *M. pulcherrima* strains representative of former species *Metschnikowia sinensis*, *Metschnikowia andauensis* and *Metschnikowia ziziphicola,* which have traits of interest such as their use as antagonistic agents (Pawlikowska et al. 2019; Horvath et al. 2021) or potential probiotic properties (Agarbati et al. 2020; 2024). The focus of the exploration was precisely the investigation of the genomic basis for their biotechnological applications and safety, adding also phenotypic evaluation, relevant for further use.

## Materials and methods

### *Metschnikowia* strains dataset, growth conditions, and DNA extraction

Genome sequencing was carried out on the strains reported in Table 1.

**Table 1:** List of the *Metschnikowia* strains considered in this study

Species	Note	NCBI Accession No	Isolation source
*Metschnikowia pulcherrima* NRRL Y-7111^ T^		JAQFHD000000000.1	Grapes, *Vitis labrusca* ‘Concord’
*Metschnikowia pulcherrima* CBS 10357	Type of the former* M. sinensis*	JAQFHA000000000.1	Jujube fruit (*Ziziphus jujuba*) surface in unmanaged orchard
*Metschnikowia pulcherrima* NRRL Y-48695	Type of the former* M. andauensis*	JAQFHC000000000.1	Gut of a caterpillar of a moth, *Helicoverpa armiger*a on corn
*Metschnikowia pulcherrima* NRRL Y-48712	Type of the former* M. ziziphicola*	JAQFHB000000000.1	Jujube fruit, *Z. jujuba*

Briefly, the total genomic DNA was isolated from 50 mL of cultures grown in YPD broth and purified using the commercial kit Wizard® SV Genomic DNA Purification System (Promega, Madison, WI, United States), following the manufacturer’s protocol. DNA concentration and purity were checked with Qubit (Life Technologies, Grand Island, NE, United States) and NanoDrop ND1000 UV–Vis Spectrophotometer (Thermo Fisher Scientific, Waltham, MA, United States), while the DNA integrity was analyzed by agarose gel electrophoresis.

### Library preparation and sequencing

The total extracted genomic DNA (1 μg) from each strain was sheared with a Covaris M220 Focused-ultrasonicator (Covaris Inc., Woburn, MA, USA) with a target size of 400 bp and used for library preparation with the Ion Xpress Plus gDNA Fragment Library kit (Life Technologies, Carlsbad, CA, United States), following the manufacturer's instructions. Library size selection (~ 400 bp) was performed by agarose gel electrophoresis using 2% E-Gel SizeSelect Agarose Gels (Life Technologies, Carlsbad, CA, United States). After purification, library concentrations were quantified using the Qubit dsDNA HS Assay Kit (Life Technologies, Carlsbad, United States). Template-positive Ion Sphere Particles were prepared for 400-base-read using the Ion OneTouch 2 System (ThermoFisher Scientific, Waltham, MA, United States) with an Ion 520 & 530 Kit-OT2 (ThermoFisher Scientific, Waltham, MA, United States) and then sequenced on an Ion 530 Chip using an Ion S5 System (ThermoFisher Scientific, Waltham, MA, United States).

### Assembly, gene prediction, and annotation

Single-end sequences were trimmed, and adapters were removed with an internal workflow of Ion Torrent Suite. Output reads were assembled with MIRA (Mimicking Intelligent Read Assembly) v5 (https://sourceforge.net/projects/mira-assembler/) (Chevreux et al. 2004), selecting specific Ion Torrent parameters. Since MIRA supports an average coverage depth of 80X, *M. pulcherrima* CBS 10357 raw reads that overcame this value were submitted to Seqtk v1.3 (https://github.com/lh3/seqtk) for a down-sampling to 5 million reads. Assembled genomes were further analysed without collapsing them into haploid genome sequences. The quality and completeness of the assembly were assessed with QUAST v5.0.2 (Gurevich et al. 2013) and BUSCO (Benchmarking Universal Single-Copy Orthologs) v5.7.1 (Manni et al. 2021). All *Metschnikowia* entire genus proteins (37756 sequences) were downloaded from NCBI and used as external evidence to annotate the genomes with BRAKER2 v2.1.5 pipeline (Bruna et al. 2021) in EP-mode with the “fungus” option activated. The genome in input for BRAKER2 was repeat masked with RepeatMasker v4.0.8 (specific library for *Metschnikowia* species) (Tarailo-Graovac and Chen 2009). Furthermore, the completeness of the prediction performed by BRAKER2 was evaluated with BUSCO (with protein mode) and general prediction statistics were calculated with Eval v2.2.8 (Keibler and Brent 2003). Proteins obtained for each sequenced strain were remapped using Miniprot (v0.13) (Li 2023) against genome assemblies recently deposited in NCBI belonging to the type-strains (NRRL Y-7111^ T^: JAJMIJ000000000.1; NRRL Y-48711 (= CBS 10357): JAJMHS000000000.1; CBS 10809 (= NRRL Y-48695): JAJMIQ000000000.1; NRRL Y-48712: JAKTYT000000000.1) (Opulente et al. 2023) with affine gap penalty, splicing and frameshift and the completeness were evaluated with BUSCO.

All the assemblies of the four *M. pulcherrima* genomes were deposited at the NCBI under the BioProject ID PRJNA906613, whereas the corresponding assembly and annotation datasets were deposited in GenBank under the accession number described in Table 1.

Predicted proteins were functionally annotated using PANNZER2 (Toronen et al. 2018), a genome-wide functional annotations web server. Moreover, PANNZER2 was used to perform functional annotations with KO (KEGG Orthology) assignments to characterize individual gene functions and reconstruct KEGG pathways. The Average Nucleotide Identity (ANI) calculation was performed using the OrthoANIu algorithm (Yoon et al. 2017) and pyani tool v. 0.2.11 (https://github.com/widdowquinn/pyani) to compare the four strains with the sequences of other *M. pulcherrima* strains deposited into NCBI (until June 2023) (FL01: VFXK00000000.1; KIOM G15050: JACBPP000000000.1; 277: ANFW00000000.2; APC 1.2: GCA_004217705.1) and other yeasts genera, chosen as outgroup (*Metschnikowia bicuspidata* var. *bicuspidata* NRRL YB-4993: JAJLWB010000000.1; *Clavispora lusitaniae* ATCC 42720: AAFT00000000.1; and *Saccharomyces cerevisiae* S288C: GCF_000146045.2). Genomes deposited by Opulente and colleagues (Opulente et al. 2023) (NRRL Y-7111^ T^: JAJMIJ000000000.1; NRRL Y-48711 (= CBS 10357): JAJMHS000000000.1; CBS 10809 (= NRRL Y-48695): JAJMIQ000000000.1; NRRL Y-48712: JAKTYT000000000.1) were used as control.

### Integrated in silico and in vitro safety assessment: focus on virulence factors, anti-fungal resistances, and biogenic amines production

To explore the potential of these strains to express fungal virulence factors, a BLASTP analysis was run on the predicted proteins against the 2,062 sequences within the Database of Fungal Virulence Factor v1.0 (DFVF) (Lu et al. 2012); the best-hits were filtered, selecting those that had an e-value less than 1e^−15^, an identity higher than 80% and a query coverage higher than 90%. The virulence factors that were eventually retrieved with high similarity to *C. lusitaniae* (i.e*.*, a pathogenic yeast responsible for human candidemia and closely related to *M. pulcherrima*) (Gabaldon et al. 2016) were further investigated.

At a phenotypic level, the Integral System Yeasts Plus kit (Liofilchem, Teramo, Italy) was employed to assess the presence of resistance patterns towards antimycotics. The panel, which consisted of wells with 12 different antimycotic compounds, was inoculated with the cell suspensions following the manufacturer’s instructions and incubated at 27 °C for 48 h. The colour changes of the medium from red to orange indicated an intermediate sensitivity, while the colour changes from red to yellow indicated a resistance behaviour. Based on the phenotypic data obtained, the sequences of ergosterol delta 5,6 desaturase (*ERG3*), and cytochrome P450 lanosterol 14α-demethylase (*ERG11*) genes of *Candida dublinensis* (*e.g*., P272L, Q160 K, H269 N, and Q327 K (Pinjon et al. 2003) and *Cryptococcus neoformans, Candida albicans* and *Candida auris* sequences (e.g., Y145 F (Sionov et al. 2012), Y140H and I471 T (Graham et al. 2021), V125 A/F126L (Williamson et al. 2022)) were used as queries and searched with a targeted BLAST analysis on the genomes.

Finally, an in-plate screening for tyrosine and histidine decarboxylase activities was performed using a modified medium, according to Gardini et al. (2006). In detail, 0.1 g of glucose, 0.06 g of bromocresol purple, 0.05 g of pyridoxal-5-phosphate (cofactor of the reaction) and 10 g of tyrosine and histidine were dissolved in 900 mL of demineralised water. After sterilisation, 100 mL of Yeast Nitrogen Base solution (6.7% w/v) without amino acids (Merck, Darmstadt, Germany), previously sterilised by filtration, were aseptically added. The final pH was adjusted aseptically to 5.3 ± 0.02 with HCl before filtration. The biogenic amine production was revealed bya violet halo surrounding the colonies. Similarly, for antimycotics, the genes linked to these biogenic amines production (tyrosine and histidine decarboxylase) were searched with a BLAST analysis against all the genomes.

### Bioinformatic prediction of protechnological features and experimental analysis of pulcherrimin production

Carbohydrate-active enzymes (CAZymes) were annotated using dbCAN2 metaserver v6.0 (Zhang et al. 2018). The predicted CAZymes were analysed with SignalP v6.0 (Teufel et al. 2022). The proteins with a signal peptide were further analysed using DeepLoc v2.0 (Thumuluri et al. 2022) to identify the subcellular localizations with a machine learning approach, focusing on the extracellular localization that could be related to protein secretion and thus eventually responsible for ecological adaptation and interaction.

As for the antagonistic activity, gene sequences responsible for proteins related to pulcherriminic acid production and transport (*pul1*, *pul2*, *pul3*, and *pul4*) were extracted, and the analysis of their flanking regions (around 30 kb) was carried out to understand their genetic configuration and to shed light on variable and reversible pulcherrimin production (Sipiczki et al. 2024). The genomes of *M. pulcherrima* 277 (ANFW00000000.2), *M. pulcherrima* KIOM G15050 (JACBPP000000000.1) and *M. pulcherrima* FL01 (VFXK00000000.1) were also included for a more comprehensive analysis.

The pulcherrimin pigment production was evaluated on YG (yeast extract, 5 g/L; glucose, 20 g/L; agar, 20 g/L; Merck, Darmstadt, Germany) plates supplemented with 0.05% (w/v) FeCl_3_ (Pawlikowska et al. 2019). After 72 h of incubation at 27 °C, the colonies were classified for their pulcherrimin production on a scale from 1 to 3 based on their colour: white colonies were scored with ‘1’, (no or very low pulcherrimin production), while dark brown colonies were scored ‘3’ (high pulcherrimin producers), colonies showing light brown intensity were scored ‘2’ (intermediate producers).

## Results

### Whole genome assembly and annotation quality assessment

The de novo genome assemblies of the four *M. pulcherrima* strains are reported in Table 2. Scaffolding ranged from a minimum of 2,373 (NRRL Y-7111^ T^) to a maximum of 4,020 scaffolds (CBS 10357) with a size ranging from 24,154,581 (NRRL Y-7111^ T^) bp to 30,852,072 bp (NRRL Y-48695) for scaffolds with a total length ≥ 1,000 bp. The difference in length with NCBI deposited genomes of the same strains (NRRL Y-7111^ T^: 15.5 Mb; CBS 10357 = NRRL Y-48711: 15.5 Mb; NRRL Y-48695 = CBS 10809: 17.93 Mb; NRRL Y-48712: 22.21 Mb) could be due to the collapsing of the latter into haploid consensus genomes assemblies (Opulente et al. 2023). This aspect could also explain some discrepancies observed for some genomes after completeness assessment by BUSCO analysis (Supplementary Material S1). The GC content spanned from 45.78% (NRRL Y-7111^ T^ and CBS 10357) to 45.82% (NRRL Y-48712), whereas N50 value spanned from 12,692 bp (CBS 10357) to 19,989 bp (NRRL Y-7111^ T^) in accordance with QUAST statistics (Table 2). Genome completeness accounted from 94.1% (CBS 10357) up to 98.2% (NRRL Y-7111^ T^), while the duplication rate ranged between 45.3% (NRRL Y-7111^ T^) and 79.9% (NRRL Y-48695) for “saccharomycetes_odb10” dataset. PANNZER2 annotations output reported from 7,572 (NRRL Y-7111^ T^) to 9,794 proteins (NRRL Y-48695) with a description, while a range from 8,137 (NRRL Y-7111^ T^) to 10,558 (CBS 10357) with at least one Gene Ontology (GO) term (Supplementary Material S2).

**Table 2:** QUAST results. All statistics are based on contigs of size >  = 500 bp, unless otherwise noted [e.g., “# contigs (> = 0 bp)” and “#Total length (> = 0 bp)” include all contigs]

	*M. pulcherrima* NRRL Y-7111^ T^	*M. pulcherrima* CBS 10357	*M. pulcherrima* NRRL Y-48695	*M. pulcherrima* NRRL Y-48712
#Contigs (> = 0 bp)	5,796	8,127	8,924	8,194
Contigs (> = 1000 bp)	2,373	4,020	3,588	3,360
Contigs (> = 5000 bp)	1,171	1,809	1,702	1,659
Contigs (> = 10,000 bp)	672	916	946	932
Contigs (> = 25,000 bp)	224	207	251	248
Contigs (> = 50,000 bp)	70	34	57	43
#Total length (> = 0 bp)	25,776,205	32,535,593	33,371,738	31,993,168
Total length (> = 1000 bp)	24,154,581	30,507,237	30,852,072	29,671,334
Total length (> = 5000 bp)	21,154,221	24,967,137	26,169,970	25,510,774
Total length (> = 10,000 bp)	17,612,331	18,614,104	20,811,394	20,283,501
Total length (> = 25,000 bp)	10,542,167	7,870,970	10,299,639	9,707,897
Total length (> = 50,000 bp)	5,251,826	2,123,772	3,745,602	2,927,373
Contigs	3,644	5,737	5,542	5,229
Largest contig	162,568	133,287	114,881	133,689
Total length	24,979,074	31,644,724	32,134,219	30,895,713
GC (%)	45.78	45.78	45.81	45.82
N50	19,989	12,692	15,085	15,285
N75	8,324	5,823	6,788	7,204
Total reads	5,847,831	5,009,648	6,438,496	6,178,225
Mapped (%)	99.73	99.69	99.77	99.78
Avg. coverage depth	64	42	53	55

The proteome completeness was estimated between 94% (CBS 10357) and 98.1% (NRRL Y-7111^ T^). The remapping by Miniprot v0.13 showed a range spanning from 99.01% (NRRL Y-48695 = CBS 10809) to 99.89% (CBS 10357 = NRRL Y-48711) of proteins. Regarding gene prediction and annotation, the number of genes varies from 10,671 (NRRL Y-7111^ T^) to 14,548 (CBS 10357) (predicted by BRAKER2 pipeline).

Other statistics on gene prediction calculated by Eval tool are reported in Supplementary Material S1).

**Fig. 1:**
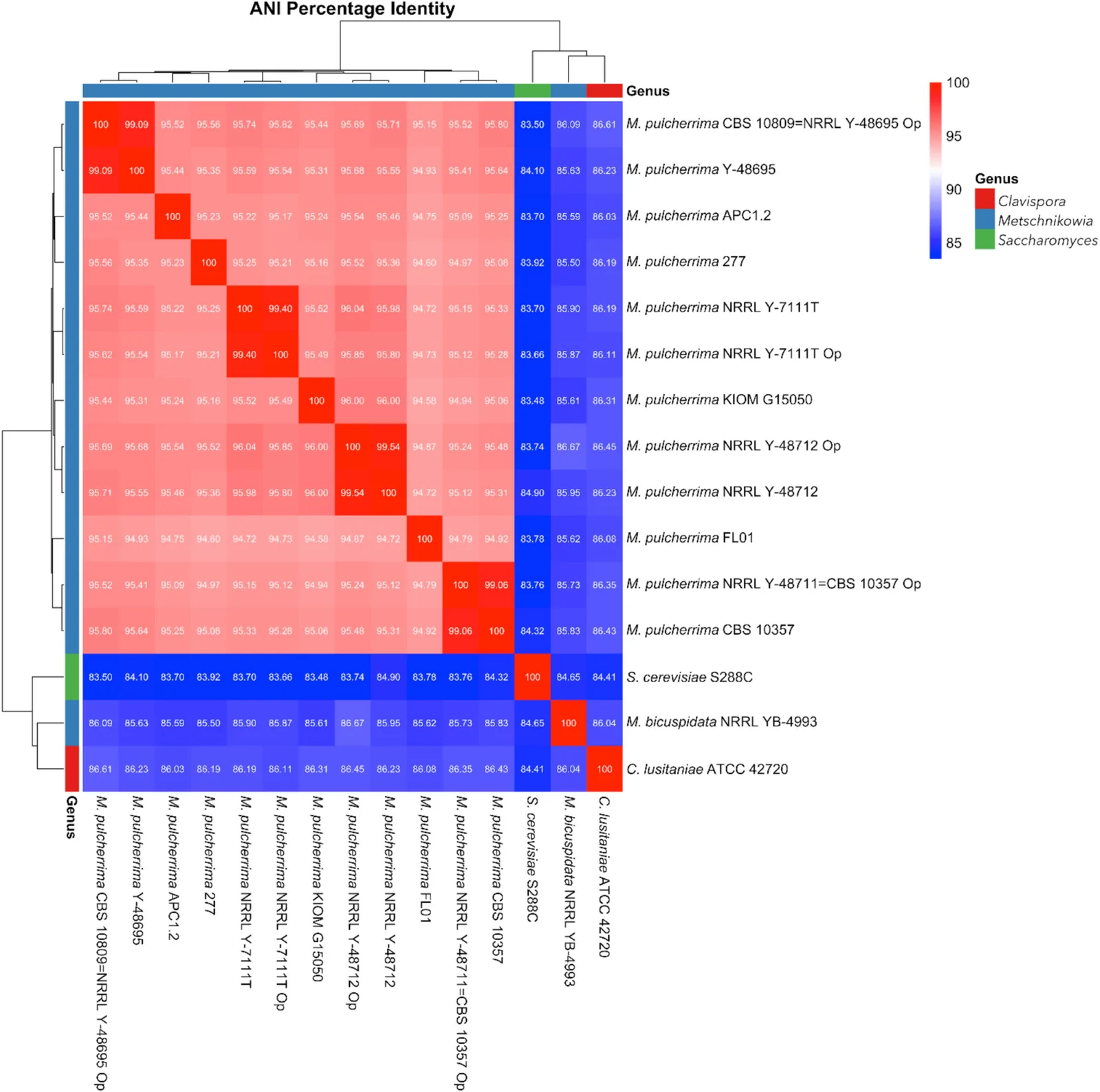
Heat map of pairwise Average Nucleotide Identity (ANI) values obtained using pyani tool v. 0.2.11. All the four analysed genomes, the already genomes of the same strains (deposited by Opulente et al. 2023, labelled with a final “op”) the available type of the former species deposited genomes until June 2023 (*M. pulcherrima* FL01* -*type of the former *‘M. citriensis'-*, *M. pulcherrima* 277 – type of the former *‘M. fructicola'—*and *M. pulcherrima* KIOM G15050 -type of the former *‘M. persimmonesis'-*, *M. pulcherrima* APC 1.2) and three outgroups (*M. bicuspidata* NRRL YB-4993, *C. lusitaniae* ATCC 42720 and *S. cerevisiae* S288 C) were compared. The values closest to 100% are in red, values closest to 90% are in white, values between 70 and 89% are in blue, and values in grey are lower than 70%

The results of ANI calculation are summarized in Fig. 1 (Supplementary Material S3). All the strains had an ANI value higher than 95%, except for *M. pulcherrima* FL01 (type of the former *M. citriensis*) (94.58–94.93%). ANI values decreased (< 87%) outside *M. pulcherrima* species. Regarding ANI values between same strains (Opulente vs this study), the values are around 99%, so this is a proof that ANI does not change using different sequencing and assembling methods. ANI calculation between the genomes obtained in the present study and the genomes deposited by Opulente was not influenced even though genome assemblies were not collapsed into haploid genome sequences (Supplementary Material S3).

### Safety assessment of the yeast strains analysed

**Fig. 2:**
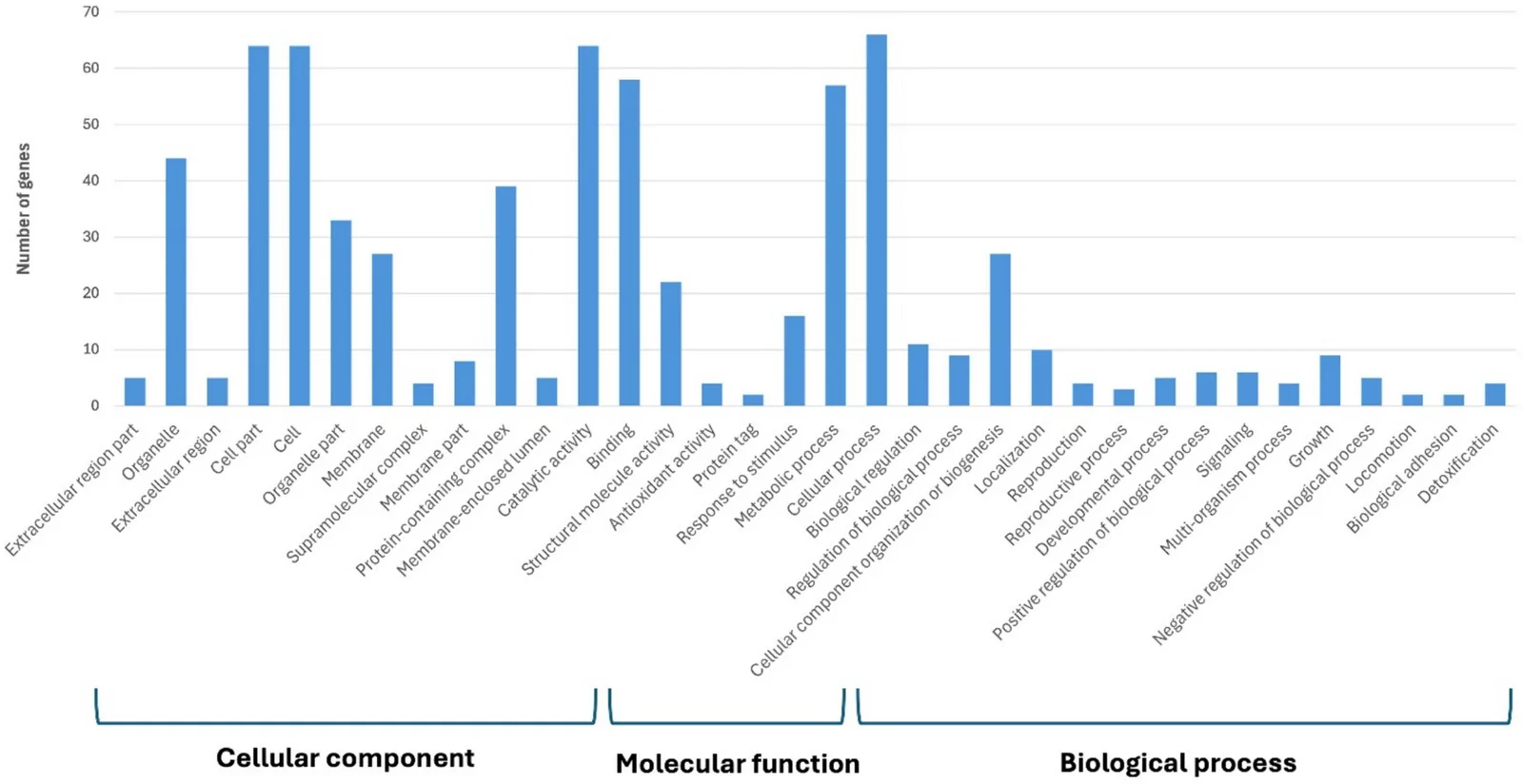
Database of Fungal Virulence Factors (DFVF) analysis for the genome dataset and GO (Gene Onthology) annotations. The bar plot displayed the percentage or the number of genes that passed the quality filter and which are annotated with Uniprot GO annotation and displayed with WEGO 2.0 tool (Ye et al. 2018)

The analysis against the DFVF (Fig. 2) revealed a similar number of genes (81, 84, 89, and 95 genes in NRRL Y-48695, NRRL Y-48712, CBS 10357 and NRRL Y-7111^ T^, respectively) putatively related to fungal virulence factors. They mainly code for enzymes responsible for glucan and chitin synthase, implicated in amino acid biosynthesis and involved in cell division or in ribosomal subunit synthesis.

Special focus was given to query F4YXH8_CLALS from *C. lusitaniae,* which is a pathogenic yeast responsible for human candidemia, and it is the most phylogenetically related to *Metschnikowia* in the so-called “GTC clade” (Gabaldon et al. 2016). The query F4YXH8_CLALS from *C. lusitaniae*, coding for a β−1,3-glucan synthase (catalytic subunit 1) and responsible for echinocandin resistance, was found in all the strains. The phenotypic resistance in *C. lusitaniae* is due to a missense mutation (S645F) in the *FKS1* gene (Desnos-Ollivier et al. 2011), which was not found in the *Metschnikowia* strains. More specifically the sequenced *M. pulcherrima* strains displayed more than one copy of gene coding for FKS1 protein which showed either a serin (S) in position 645 (similar to the non-pathogenic *C. lusitaniae* wild type) or a proline (P) (Supplementary Material S4).

At the phenotypic level, an intermediate sensitivity for itraconazole (1 µg/mL) was detected in all the strains under analysis (Supplementary Material S5), which led to a deeper investigation of genes coding for the ergosterol delta 5,6 desaturase (ERG3) and cytochrome P450 lanosterol 14 and α-demethylase (ERG11) as putative traits responsible for this phenotype due to point mutations (Williamson et al. 2022; Graham et al. 2021; Sionov et al. 2012; Pinjon et al. 2003). In the comparisons performed, no mutations associated with the resistance were retrieved (Supplementary Material S6). As for biogenic amines detection, no positive matches were retrieved from tyrosine decarboxylase and histidine decarboxylase gene search, and none of the colonies showed a positive reaction (violet halos) (Supplementary Material S7) confirming the genotypic observations.

### Bioinformatic analysis and experimental lab validation of biocontrol features

**Fig. 3:**
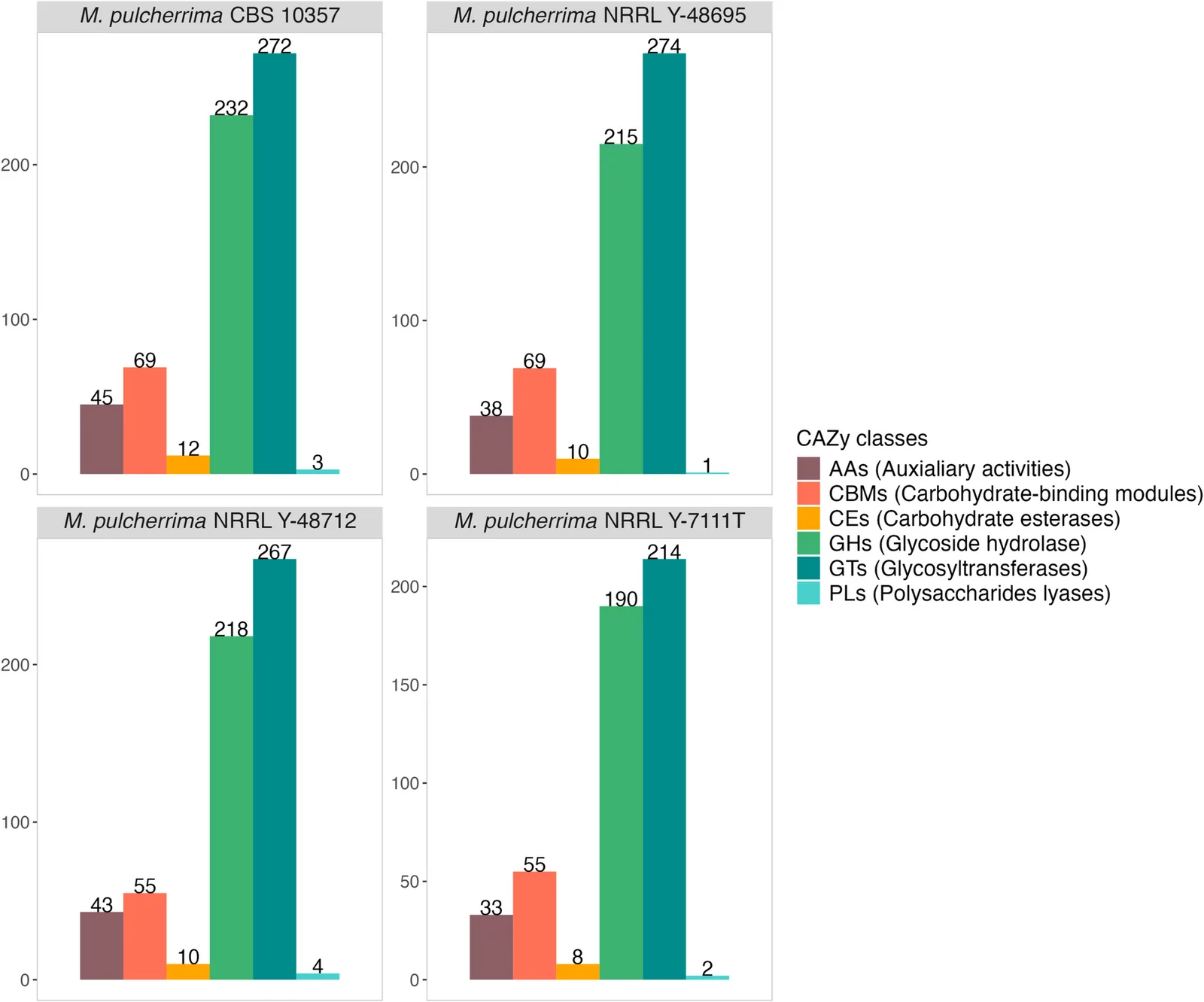
Bar plots representing the analysis of CAZymes (carbohydrate-active enzymes) on the four genomes of *Metschnikowia pulcherrima* sequenced in this study. The colours of bars displayed in the plot are related to CAZy classes (AAs: Auxiliary activities [dark brown]; CBMs: carbohydrate-binding modules [red]; CEs: carbohydrate esterarases [yellow]; GHs: glycoside hydrolases [light green]; GTs: glycosyl transferases [dark green]; PLs:polysaccharides lyases [cyan]). The number of hits for each class is reported on the top of the bar

The dbCAN metaserver annotated 633 CAZymes for CBS 10357, 502 for NRRL Y-7111^ T^, 607 for NRRL Y-48695 and 597 for NRRL Y-48712 (Fig. 3), among which the glucoside hydrolases (GHs) and the glycosyl transferases (GTs) were the most represented.

**Fig. 4:**
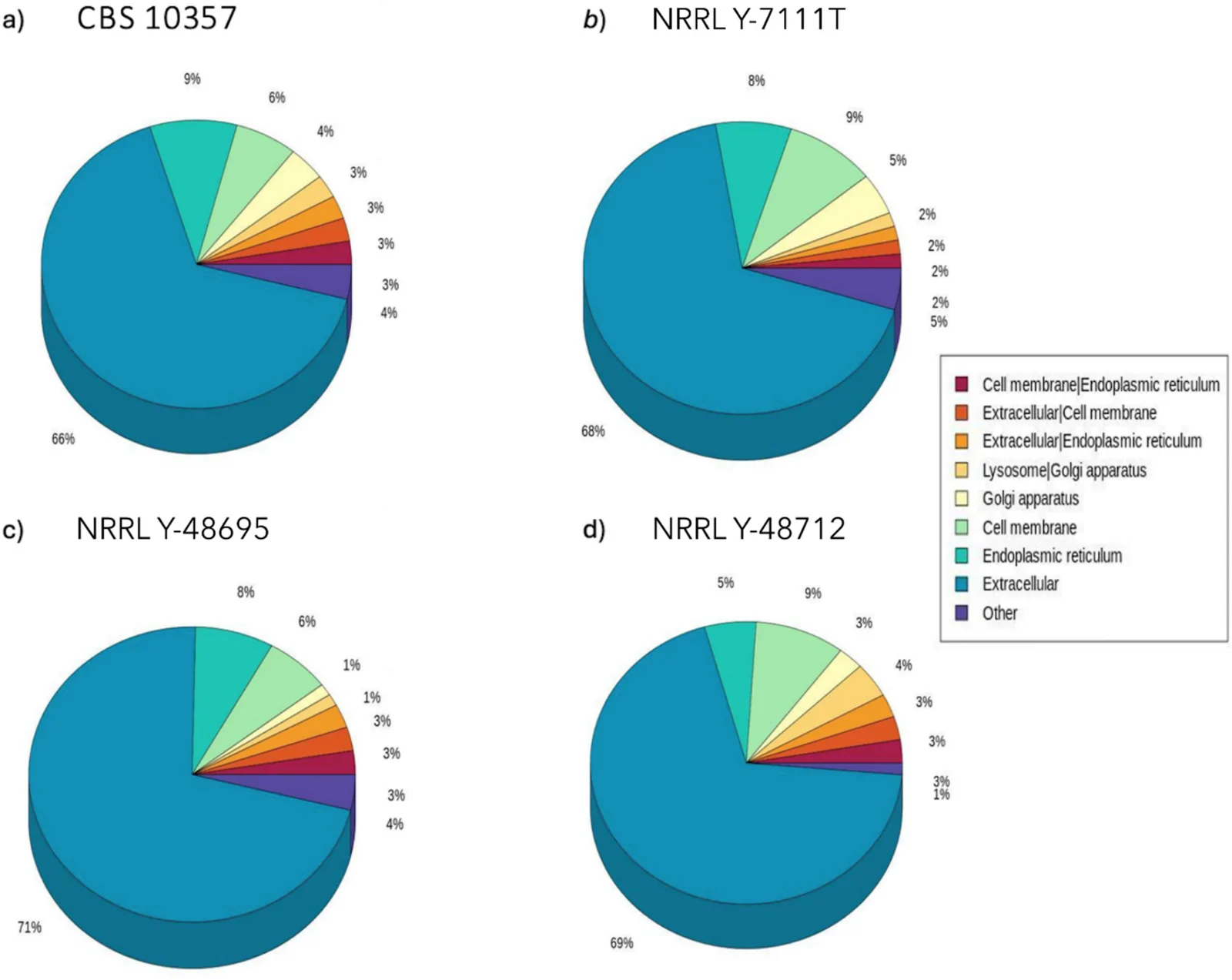
Distribution of CAZymes with a signal peptide based on their subcellular localisation for each strain: **a**) *M. pulcherrima* CBS 10357, **b**) *M. pulcherrima* NRRL Y-7111.^T^, **c**) *M. pulcherrima* NRRL Y-48695, **d**) *M. pulcherrima* NRRL Y-48712

A signal peptide was predicted in 12–13% of the CAZymes in all the genomes, indicating that these enzymes could be secreted and exert an activity outside the cell (Table 3); as shown in Fig. 4, DeepLoc v2.0 analysis revealed that the majority of them had an extracellular localisation ranging from 66% up to 71% for CBS 10357 and NRRL Y-48695, respectively.

**Table 3:** Summary of dbCAN and SignalP v6.0 output. For each strain the number of CAZymes and the number of CAZymes with a signal peptide predicted are displayed. The percentages are referred to the total predicted CAZymes

Strain	Number of CAZymes (dbCAN)	Number of CAZymes with a signal peptides (SignalP) (% on the total CAZymes)
*M. pulcherrima* CBS 10357	633	77 (12%)
*M. pulcherrima* NRRL Y-7111^ T^	502	65 (13%)
*M. pulcherrima* NRRL Y-48695	607	77 (13%)
*M. pulcherrima* NRRL Y-48712	597	75 (13%)

This subset of extracellular CAZymes was further investigated for their potential role in biocontrol activity. The list of the extracellular CAZymes classes is shown in Table 4. Several GH-class enzymes are represented in the genomes multiple times, probably due to heterozygous chimeric genomes. The majority of predicted extracellular CAZymes are present in all the four strains, except for GH65 coding for an α,α-trehalase that is specific for the strain NRRL Y-48695, GH31 coding for α-glucosidase specific for NRRL Y-7111^ T^ and GH47 coding for α-mannosidase predicted only in CBS 10357 and NRRL Y-48712 strains.

**Table 4:** Extracellular CAZymes classes predicted in the four *Metschnikowia pulcherrima* genomes using the combined approach with dbCAN, SignalP v6.0, and DeepLoc v2.0

CAZy_class	EC number	CAZymes	Strains	Gene number and locus tags
AA1	1.10.3.2	Laccase/*p*-diphenol:oxygen oxidoreductase/ferroxidase	All	**NRRL Y-48695**: (3) g1991.t1, g6101.t1, g5255.t1 **NRRL Y-7111**^**T**^: (1) g6735.t1 **CBS 10357:** (2) g217.t1, g2972.t1 **NRRL Y-48712:** (2) g4218.t1, g8857.t1
CBM18 + GH16_19	-	Modules of approx. 40 residues. The chitin-binding function has been demonstrated in many cases. These modules are found attached to a number of chitinase catalytic domains but also in non-catalytic proteins either in isolation or as multiple repeats and xyloglucan:xyloglucosyltransferase	All	**NRRL Y-48695:** (4) g5860.t1, g5861.t1, g9743.t1, g9778.t1 **NRRL Y-7111**^**T**^: (2) g4857.t1, g4858.t1 **CBS 10357**: (4) g11012.t1, g7143.t1, g7931.t1, g7932.t1 **NRRL Y-48712**: (3) g13478.t1, g6388.t1, g8607.t1
CBM43 + GH72	2.4.1.-	Modules of approx. 90–100 residues were found at the C-terminus of GH17 or GH72 enzymatic modules and were sometimes isolated. CBM43 modules sometimes carry a C-terminal membrane anchor. + β−1,3-glucanosyltransglycosylase	All	**NRRL Y-48695:** (3) g703.t1, g8464.t1, g9577.t1 **NRRL Y-7111**^**T**^: (1) g6636.t1 **CBS 10357:** (2) g13685.t1, g5358.t1 **NRRL Y-48712:** (3) g4055.t1, g4641.t1, g7513.t1
GH132	3.2.1.-	Activity on β−1,3-glucan (curdlan) shown for the *Aspergillus fumigatus* Sun4 protein; activity on laminarioligosaccharides shown for *A. fumigatus* Sun4 protein and *Candida albicans* Sun41 protein; transglycosylation activity reported in Gastebois et al. (2013); β−1,3-glucosidase (EC 3.2.1.-)	All	**NRRL Y-48695:** (5) g10677.t1, g2637.t1, g4470.t1, g490.t1, g521.t1 **NRRL Y-7111**^**T**^: (4) g2266.t1, g3316.t1, g4436.t1, g7878.t1 **CBS 10357:** (6) g12973.t1, g1648.t1, g2406.t1, g244.t1, g2650.t1, g8723.t1 **NRRL Y-48712:** (6) g10658.t1, g12769.t1, g6390.t1, g6899.t1, g8344.t1, g882.t1
GH16_18	2.4.1.-|3.2.1.39	Chitin β−1,6-glucanosyltransferase/endo-1,3-β-glucanase/laminarinase	All	**NRRL Y-48695:** (2) g8750.t1, g9012.t1 **NRRL Y-7111**^**T**^: (2) g362.t1, g9751.t1 **CBS 10357:** (2) g13021.t1, g5237.t1 **NRRL Y-48712:** (2) g1195.t1, g7227.t1
GH17	3.2.1.39|3.2.1.-	Glucan endo-1,3-β-glucosidase/β−1,3-glucosidase	All	**NRRL Y-48695:** (9) g1049.t1, g11466.t1, g3739.t1, g3743.t1, g7313.t1, g13438.t1, g13494.t1, g8203.t1 **NRRL Y-7111**^**T**^: (8) g107.t1, g1406.t1, g4337.t1, g6168.t1, g704.t1, g7971.t1, g9163.t1, g9145.t1 **CBS 10357:** (5) g11118.t1, g2150.t1, g8279.t1, g9029.t1, g13722.t1 **NRRL Y-48712:** (7) g11229.t1, g1226.t1, g1669.t1, g2566.t1, g5280.t1, g717.t1, g8631.t1
GH18	3.2.1.14	Chitinase	All	**NRRL Y-48695:** (6) g2289.t1, g4211.t1, g6874.t1, g7843.t1, g6873.t1, g4212.t1 **NRRL Y-7111**^**T**^: (6) g1937.t1, g3685.t1, g9305.t1, g9835.t1, g9304.t1, g3686.t1 **CBS 10357:** (6) g3157.t1, g3158.t1, g4751.t1, g5535.t1, g3156.t1, g4750.t1 **NRRL Y-48712:** (6) g5711.t1, g5712.t1, g827.t1, g828.t1, g5713.t1, g826.t1
GH5_9	3.2.1.58|3.2.1.21|3.2.1.75	Glucan β−1,3-glucosidase (EC 3.2.1.58), β-glucosidase (EC 3.2.1.21), glucan endo-1,6-β-glucosidase (EC 3.2.1.75)	All	**NRRL Y-48695:** (3) g11676.t1, g1471.t1, g9322.t1 **NRRL Y-7111**^**T**^: (3) g4029.t1, g4895.t1, g6015.t1 **CBS 10357:** (4) g12636.t1, g3119.t1, g5107.t1, g5966.t1 **NRRL Y-48712:** (4) g11533.t1, g6183.t1, g8466.t1, g8990.t1

**Fig. 5:**
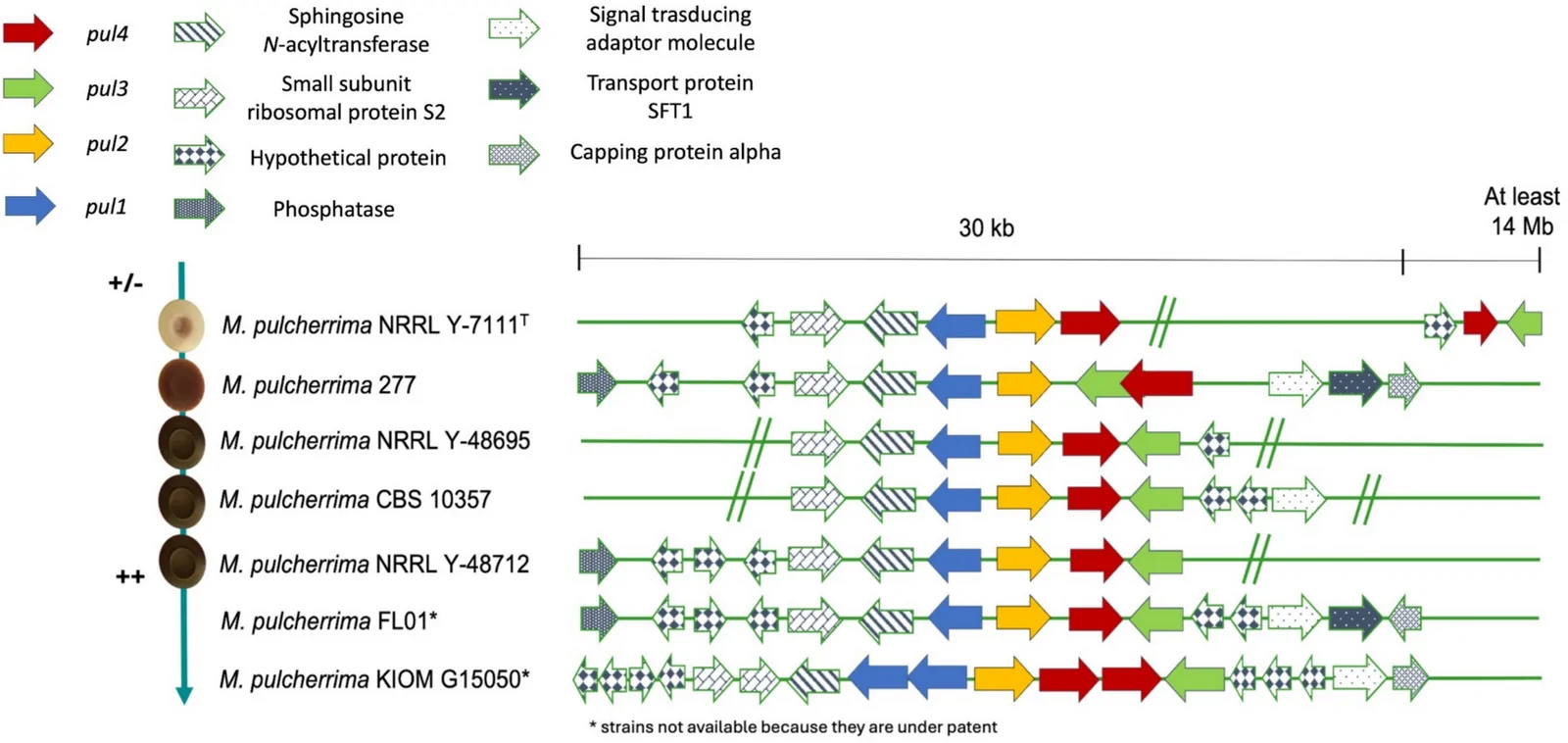
Schematic representation of pulcherrimin genes cluster configuration of the four genomes analyzed associated with pulcherrimin phenotype. The legend shows color code for represented genes. On the left, pulcherrimin phenotype is shown as red colony pigmentation associated to each strain. For FL01 and KIOM G15050 strains pulcherrimin phenotypes are not available because they are under patent (*). “//” points out the end of the contig

Regarding the genes responsible for the production of pulcherrimin and its transport (*pul* genes), at least two copies per strain for each *pul* gene were found, except for *M. pulcherrima* KIOM G15050 (type of the former *M. persimmonesis*). Most of the genomes displayed the *pul* genes arranged as *pul1*-*pul2*-*pul4*-*pul3*, as shown in Fig. 5 (only one chromosome was displayed). Differently, in *M. pulcherrima* NRRL Y-7111^ T^ although *pul1*, *pul2* and *pul4* were syntenic, another copy of *pul4 *and *pul3* were mapped in a different contig. Regarding M. *pulcherrima* 277, the *pul3* gene was located between *pul2* and *pul4.*

The analysis of the flanking regions (about 30 kb upstream and downstream) of the *pul* cluster showed that all the strains displayed almost the same traits: genes coding for a sphingosine *N*-acyltransferase, the small subunit ribosomal protein S2 and from one to four hypothetical proteins were detected upstream *pul1*, while downstream *pul3*, the signal transducing adaptor molecule and a transport protein SFT1 were observed (except for *M. pulcherrima* NRR Y-7111^ T^ and *M. pulcherrima* 277).

Moreover, based on the prediction of protein localization performed by DeepLoc v2.0, Pul1 and Pul4 proteins seem to be located in the cytoplasm or in the nucleus, Pul2 is in the endoplasmic reticulum, Pul3 in the cell membrane and SNF2, a transcriptional regulator involved in pulcherrimin production, is in the nucleus for all the strains (Table 5). The predicted localizations of these proteins reflected their functions, as Pul1 and Pul2 are involved in the pulcherriminic acid synthesis, Pul3 is a transporter, Pul4 and SNF2 are transcription factors that regulate the process.

**Table 5:** DeepLoc v2.0 analysis on pulcherrimin genes (pul). The table showed the predicted subcellular localisation for Pul1, Pul2, Pul3, Pul4 and SNF2 proteins for the four strains analysed and the genes'names are in brackets

	Pul1	Pul2	Pul3	Pul4	SNF2
*M. pulcherrima* NRRL Y-7111^ T^	Cytoplasm| nucleus (g6234.t1, g1414.t1)	Endoplasmic reticulum (g1413.t1, g6235.t1)	Cell membrane (g7015.t1, g7488.t1)	Cytoplasm| nucleusnucleus (g7014.t1, g9796.t1, g1412.t1, g6236.t1)	Nucleus cytoplasm| nucleus (g9093.t1, g315.t1, g314.t1, g9682.t1, g6750.t1)
*M. pulcherrima* CBS 10357	Cytoplasm| nucleus (g1755.t1, g2887.t1)	Endoplasmic reticulum (g1756.t1, g2886.t1)	Endoplasmic reticulum cell membrane (g1758.t1, g2884.t1)	Cytoplasm| nucleus (g2885.t1, g1757.t1)	Nucleus (g3366.t1, g6146.t1, g7894.t1)
*M. pulcherrima* NRRL Y-48695	Cytoplasm| Nucleus (g5462.t1, g3339.t1)	Endoplasmic reticulum (g5461.t1, g3340.t1)	Cell membrane (g5459.t1, g952.t1)	Cytoplasm| nucleus (g953.t1, g5460.t1)	Nucleus cytoplasm| nucleus (g11414.t1, g9613.t1, g5612.t1, g9971.t1, g14010.t1, g12435.t1)
*M. pulcherrima* NRRL Y-48712	Cytoplasm| nucleus (g8578.t1, g4686.t1)	Endoplasmic reticulum (g4685.t1, g8579.t1)	Cell membrane (g8581.t1, g4683.t1)	Cytoplasm| nucleus (g8580.t1, g4684.t1)	Nucleus extracellular (g6846.t1, g6260.t1, g9877.t1, g13287.t1)

As for the pulcherrimin production phenotypic validation, NRRL Y-7111^ T^ colonies were white-cream with pinkish streaks, then classified as low producer, whereas NRRL Y-48695, NRRL Y-48712 and CBS 10357 colonies appeared dark brown, indicating high production. Moreover, *M. pulcherrima* 277 (a type of the former *M. fructicola*) showed light brown colonies and was classified as a medium pulcherrimin producer (Fig. 5 and Supplementary Material S8).

## Discussion

The exploration of the non-conventional yeast potential is among the relevant trends in the field of yeast biotechnology and in the development of bio-based green solutions (Geijer et al. 2022). The limited “omics” information for these bioresources represents one of the bottlenecks for the development of a robust body of knowledge necessary for promoting innovation in this field and assessing their safety, both for operators and end-users (Geijer et al. 2022). In the wake of these trends, here we sequenced the genomes of four strains of *M. pulcherrima*, improving the biological information on a species that, especially in the light of the reclassification conducted in 2022 (Sipiczki 2022b), appears to be the most valued one for this genus.

The sequencing of the genomes provided high-quality data both in terms of completeness and quality of the assembly: the number of contigs obtained for each assembly was less than 1000, and a completeness higher than 90% (94–98%, based on number of matches to BUSCO selected gene sets) was obtained as recommended by EFSA (2024). Completeness values were also higher compared to the genome assemblies deposited in NCBI (from July 2023) of the same strains obtained with Illumina sequencing, which span from 67.5% (NRRL Y-48712) to 97.6% (NRRL Y-7111^ T^), with a lower level of duplicated genes (Supplementary Material S1). Further, Illumina-sequenced strains (except for NRRL Y-7111^ T^) showed a generally higher number of scaffolds (up to 10,000) and lower N50 values (2–4 kb in NRRL –Y-48695 and NRRLY48712) (Opulente et al. 2023). These data suggest that genomes sequenced with Ion Torrent seem to better represent the high intragenomic differences and genome chimerisation suggested by Sipiczki and colleagues (Sipiczki et al. 2024). Further, genomic information seems to be more preserved compared to the Illumina -sequenced genomes as shown by BUSCO and Miniprot remapping.

As for genome-based strain identification, the ANI calculation confirmed that these strains belong to the species *M. pulcherrima*, as ANI values were higher than 95%, the threshold proposed for yeast species delineation (95 ± 0.5%; (Libkind et al. 2020). These data once again substantiate the reclassification of the species within the *M. pulcherrima* clade in *M. pulcherrima* (Sipiczki 2022b), which was also supported by Troiano et al. (2023) and further recommended the use of ANI for yeast identification, as already suggested in previous studies (Cortimiglia et al. 2024; Libkind et al. 2020; Lachance et al. 2020).

Obtained genome sequences were first investigated to assess the safety of these strains, an essential prerequisite for their applicability. This aspect is necessary since drug-resistant fungal infections pose a growing global health threat, with increasing cases of severe mycoses and related mortality rates (Vitiello et al. 2023). In the present study, data obtained from the interrogation of Fungal Virulence Factor Database did not reveal a peculiar capability of the four strains to produce virulence factors, as most of the genes retrieved were also involved in growth and development, and none of them was directly linked to a disease progression (Van de Wouw and Howlett 2011). As for anti-fungal resistance, no databases have been specifically developed for the detection of antimycotic resistance genes, but the phenotypic assay conducted showed only an intermediate resistance to itraconazole which, however, did not find a clear match at the genotypic level. As for biogenic amines, none of the strains were able to produce these compounds.

Safety-related data agree with the recent literature where, to date, only one strain of *M. pulcherrima* was described to cause illness in a compromised patient (Mohl et al. 1998), while very few studies report the ability of *M. pulcherrima* strains (isolated from wine) to produce biogenic amines (Tristezza et al. 2013; Staniszewski and Kordowska-Wiater 2023). In a recent study (Rahmat et al. 2024), toxicity evaluation of extracts of *M. pulcherrima* strains (belonging to former *M. persimmonesis*) showed that they did not exert harmful effects on the liver and mitochondria of zebrafish and no potential risk of cardiotoxicity was observed in hERG-HEK293 cell lines; further, other strains of *M. pulcherrima* (formerly identified as *M. ziziphicola*), selected as potential probiotics, did not show any hemolytic activity (Staniszewski and Kordowska-Wiater 2023).

Even though the safety assessment has been recently conducted for yeast strains used in food and feed, safety analysis of yeasts is still under consideration since protocols are less developed than bacterial assessments, and no standardised methods have been established yet. According to EFSA, genome sequences should be searched to identify the presence/absence of metabolic pathways involved in toxigenicity or resistance to antimycotics and, if detected, proper analysis are required to validate the in silico evidences (EFSA et al. 2018). In this framework, the present work not only unravels the safety of *M. pulcherrima* strains, considering both genotypic as well as phenotypic investigation of safety-related traits but also lays the groundmark for genome-based safety analysis of yeasts used in fermented beverages or for biocontrol in agriculture.

In terms of biotechnological potential, the strains in the present study were isolated from plants/fruits (NRRL Y-7111^ T^; CBS 10357, NRRL Y-48712) and from plant-associated insect gut (NRRL Y-48695) (Table 1). The carbohydrate-active enzymes (CAZymes) prediction reflects the capability of these strains to colonize plant surfaces or insect guts and their potential as biocontrol agents. The enzymes found in the four strains under analysis, spanning from 502 in *M. pulcherrima* NRRL Y-7111^ T^ to 633 in NRRL Y-48712, seem to hold a high specificity for plant-surface, especially for the class glycoside hydrolases (GH). Several of them could also be involved in fungal cell wall degradation, as hypothesized for the CAZymes of *M. pulcherrima* 277 (Piombo et al. 2018). Particular attention was given to those CAZymes for which a signal peptide was predicted, indicating a putative extracellular localization. Signal peptides, in fact, play a crucial role in protein secretion, making them interesting elements for biotechnological applications, including the development of biological control agents (Thak et al. 2020). In this perspective, Jones and Prusky (2002) demonstrated the potential of expressing anti-fungal peptides in yeast using signal peptides for secretion, offering a novel approach to control postharvest diseases. For example, chitinase (GH18) activity may contribute to the antagonistic effect of *M*. *pulcherrima* species, as it was already observed for *M. fructicola* AP47, which showed a higher transcriptional intensity of the chitinase gene in the presence of fungal pathogen *Monilinia fructicola* cell wall (Banani et al. 2015). Furthermore, α-mannanase (GH76) activity, for instance, was also detected in a *Salegentibacter* sp. with the ability to consume alpha-mannan from fungi (Solanki et al. 2022), while β−1,3-glucanase (GH81) was identified in *Clostridium* strains with the ability to kill the plant pathogen *Fusarium oxysporum* (Ueki et al. 2020). About environmental adaptation, the α,α-trehalase (GH65), found only in NRRL Y-48695 strain, seems essential for sex pheromone biosynthesis in *Helicoverpa armigera* (Zhang et al. 2022), which is also the host of this strain, clearly indicating the adaptation of this microorganism to the insect gut environment.

As for pulcherrimin production, both genotypic and phenotypic analysis confirmed the ability of these strains to produce this pigment, but differences in colony pigmentation were also observed, which seem not to be related to a specific genomic structure. In accordance with Sipiczki et al. (2024), upon longer periods of incubation (around 3 weeks or more), a mixed population of colonies of different color intensities can be observed, indicating an instability and reversibility in pulcherrimin production. This evidence, combined with the different number of copies of *pul* genes clusters, could validate the hypothesis of epigenetic processes at the basis of pulcherrimin production (Sipiczki et al. 2024).

## Conclusions

The results obtained in this study clearly showed that the availability of genomic sequences could be relevant resources i) to characterise a new strain or to confirm a new yeast species description, ii) to open the possibility of performing a genome-based phenotype exploration for traits of interest in the development of bio-based solutions, iii) and to assess the safety of the strain, to exclude the spreading of anti-fungal resistance in food and feed chains or any other risks for consumers and stakeholders. Our findings suggest that governmental authorities, such as EFSA, should update guidelines, defining standardized protocols to assess safety for yeast species as already done for bacteria. In this regard, the paper contributes to a general pipeline for genomic characterisation and safety assessment of unconventional yeasts, using *M. pulcherrima* as a model species.

## Supplementary Information

Below is the link to the electronic supplementary material.

Supplementary file1 (DOCX 9644 KB)

## Data Availability

All the assemblies of the four *Metschnikowia* *pulcherrima* genomes were deposited at the NCBI under the BioProject ID PRJNA906613, whereas the corresponding assembly and annotation datasets were deposited at DDBJ/ENA/GenBank under the accession number JAQFHD000000000.1 (NRRL Y-7111 T); JAQFHA000000000.1 (CBS 10357); JAQFHC000000000.1 (NRRL Y-48695) and JAQFHB000000000.1 (NRRL Y-48712).
