# Supplementary material for: Unlocking the potential of Metschnikowia pulcherrima: a dive into the genomic and safety characterization of four plant-associated strains
Source: Appl Microbiol Biotechnol. 2025 May 29;109:128. doi: 10.1007/s00253-025-13515-0 (PMC12122572; doi:10.1007/s00253-025-13515-0)
Supplement: Supplementary file 1 [file 253_2025_13515_MOESM1_ESM.docx]

**Unlocking the potential of *Metschnikowia* *pulcherrima*: a dive into the genomic and safety characterization of four plant-associated strains**

Ilaria Larini^1§^, Massimo Ferrara^2§^, Eleonora Troiano^1^, Veronica Gatto^1^, Giuseppina Mulè^2^, Nicola Vitulo^1^, Vittorio Capozzi^3^*, Elisa Salvetti^1,4^*, Giovanna E. Felis^1,4^**, Sandra Torriani^1^**

Submitted to: **Applied Microbiology and Biotechnology**

**SUPPLEMENTARY MATERIAL**

**Supplementary Material S2.** Stacked bar plot representing genome assemblies completeness based on the BUSCO metrics. Genes predicted as Complete (splitted in Single-copy (S) and Duplicated (D)), Fragmented (F) and Missing (M) were are displayed as percentage. Strains labelled with “A” have been sequenced in this study and strains labelled with “B” were retrieved from NCBI (Opulente et al. 2023).

**Supplementary Material S1**. General statistics of genome annotation related to a) *M. pulcherrima* CBS 10357; b) *M. pulcherrima* NRRL Y-7111T; c) *M. pulcherrima* NRRL Y-48695; d) *M. pulcherrima* NRRL Y-48712.

a)

| *M. pulcherrima* CBS 10357 | Genes | Transcripts | Exons | Introns |
| --- | --- | --- | --- | --- |
| Count | 14548 | 14897 | 15317 | 1159 |
| Average length | - | 1207.55 | 1133.61 | 168.68 |
| Median length | - | 978 | 921 | 99 |
| Total length | - | 17988924 | 17363536 | 195500 |
| Average coding length | - | 1194.37 | - | - |
| Median coding length | - | 966 | - | - |
| Total coding length | - | 17792458 | - | - |
| Average score | - | 1.01 | 0.94 | 0 |
| Total score | - | 15028.74 | 14433.28 | 0 |
| Ave exons per | - | 1.08 | - | - |
| Med exons per | - | 1.00 | - | - |
| Total transcripts | 14897 | - | - | - |
| Transcripts per | 1.02 | - | - | - |

b)

| *M. pulcherrima* NRRL Y-7111^T^ | Genes | Transcripts | Exons | Introns |
| --- | --- | --- | --- | --- |
| Count | 10671 | 10893 | 11513 | 841 |
| Average length | - | 1303.37 | 1206.30 | 176.77 |
| Median length | - | 1059.00 | 975 | 104 |
| Total length | - | 14197645 | 13888102 | 148662 |
| Average coding length | - | 1289.68 | - | - |
| Median coding length | - | 1047 | - | - |
| Total coding length | - | 14048454 | - | - |
| Average score | - | 1.04 | 0.96 | 0 |
| Total score | - | 11304.10 | 11095.12 | 0 |
| Ave exons per | - | 1.08 | - | - |
| Med exons per | - | 1.00 | - | - |
| Total transcripts | 10893 | - | - | - |
| Transcripts per | 1.02 | - | - | - |

c)

| *M. pulcherrima* NRRL Y-48695 | Genes | Transcripts | Exons | Introns |
| --- | --- | --- | --- | --- |
| Count | 14389 | 14685 | 15141 | 1035 |
| Average length | - | 1235.78 | 1162.31 | 166.63 |
| Median length | - | 1008 | 946 | 105 |
| Total length | - | 18147397 | 17598534 | 172467 |
| Average coding length | - | 1223.88 | - | - |
| Median coding length | - | 993 | - | - |
| Total coding length | - | 17972653 | - | - |
| Average score | - | 1.02 | 0.95 | 0 |
| Total score | - | 14995.07 | 14445.94 | 0 |
| Ave exons per | - | 1.07 | - | - |
| Med exons per | - | 1.00 | - | - |
| Total transcripts | 14685 | - | - | - |
| Transcripts per | 1.02 | - | - | - |

d)

| *M. pulcherrima* NRRL Y-48712 | Genes | Transcripts | Exons | Introns |
| --- | --- | --- | --- | --- |
| Count | 13904 | 14157 | 14560 | 1079 |
| Average length | - | 1246.57 | 1170.82 | 165.85 |
| Median length | - | 1011 | 954 | 107 |
| Total length | - | 17647739 | 17047086 | 178948 |
| Average coding length | - | 1233.78 | - | - |
| Median coding length | - | 999 | - | - |
| Total coding length | - | 17466646 | - | - |
| Average score | - | 1.01 | 0.94 | 0 |
| Total score | - | 14269.40 | 13742.98 | 0 |
| Ave exons per | - | 1.08 | - | - |
| Med exons per | - | 1.00 | - | - |
| Total transcripts | 14157 | - | - | - |
| Transcripts per | 1.02 | - | - | - |

**Supplementary Material S3**. Heatmap matrix of Average Nucleotide Identity (ANI) comparison. The analysis includes the four analysed genomes, the available former type deposited genomes (*M. pulcherrima* FL01 -former type of ‘*M. citriensis'*-, *M*. *pulcherrima* 277 – former type of ‘*M. fructicola*' - and *M. pulcherrima* KIOM G15050 – former type of ‘*M*. *persimmonesis'*-, *M. pulcherrima* APC 1.2), the available *M. pulcherrima* genomes deposited by Opulente et al., 2023 of the same strains sequenced in this study (JAJMIQ010000001.1 = CBS 10809; JAJMHS010000001.1 = NRRL Y-48711; JAKTYT010000001.1 = NRRL Y-48712; JAJMIJ010000001.1 = NRRL Y-7111T)and three outgroups (*M. bicuspidata* NRRL Y-4993, *C. lusitaniae* ATCC 42720 and *S. cerevisiae* S288C). The comparison between Opulente genomes and genomes of this study are highlighted in colors.

**Supplementary Material S4.** FKS1 protein alignment for the four strain of *M. pulcherrima* under analysis in this study (NRRL Y-7111T, CBS 10357, NRRL Y-48695 and NRRL Y-48712) and F4YXH8_CLALS (wild-type) and AEC04742.1 (with S645F mutation) strains belonging from *Clavispora* *lusitaniae* strains. The position of the mutation (S645F) was highlighted in green and the region HS1 in which it occurred is in red.

**Supplementary Material S5** **–** Integral System Yeasts Plus panel (Liofilchem, Teramo, Italy) used for antifungal resistance test of NRRL Y-48695 strain. The assay includes 24 reaction well panel containing biochemical substrates and antimycotics. Wells from 1 to 13 refer to the assimilation of various sugars (yellow = positive; purple = negative); well 14 to 23 refer to wells containing antimycotics (red = sensible to the antimycotic; yellow = resistant to the antimycotic; orange = intermediate sensitivity). The 24^th^ well refers to yeast growth test (yellow = positive; purple = negative). Antimycotics concentrations are: 1.25 µg/mL Nystatin (NY); 2 µg/mL Amphotericin (AMB); 16 µg/mL Flucytosine (FCY); 2 µg/mL Econazole (ECN); 0.5 µg/mL Ketoconazole (KCA); 1 µg/mL Clotrimoxazole (CLO); 2 µg/mL Miconazole (MIC); 1 µg/mL Itraconazole (ITR); 2 µg/mL Voriconazole (VOR); 64 µg/mL Fluconazole (FLU).

**Supplementary Material S6.** Sequence alignment of ergosterol delta 5,6 desaturase (ERG3) and cytochrome P450 lanosterol 14α-demethylase (ERG11) proteins belonging to *M. pulcherrima* strain analyzed. *Candida dublinensis* sequence (CAD13131.1) for ERG3 and *Cryptococcus neoformans* (AEX20236.1; AEX20237.1), *Candida albicans* (AAW50593.1) *Candida auris* (UVT84711.1; UNE56009.1) and *S. cerevisiae* (QHB08993.1; PDB accession: 7RY8; 7RY9; 7RYA; 7RYB) sequences for ERG11 were used as reference.

**Supplementary Material S7.** In-plate assay for biogenic amines production (tyrosine decarboxylase and histidine decarboxylase activities) used for the screening of *M. pulcherrima* NRRL-Y48695, *M. pulcherrima* NRRL Y-48712, *M. pulcherrima* NRRL Y-7111, *M. pulcherrima* CBS 10357, *M. pulcherrima* 277. The test was conducted using Gardini et al. (2006). The absence of a halo surrounding colonies corresponds to negative results.

**277**

**CBS 10357**

**NRRL Y-7111**

**NRRL Y-48695**

**NRRL Y-48712**

**NRRL Y-48712**

**NRRL Y-48695**

**CBS 10357**

**NRRL Y-7111**

**277**

**Supplementary Material S8.**  Pulcherrimin production test on the plate of the strain analysed and M. pulcherrima 277 (type of the former M. fructicola 277). The test was carried out using YG agar supplemented with Fe(III) ions; the tested strains were spotted in the following order clockwise from creamy-white colony: M. pulcherrima NRRL Y-7111^T^, M. pulcherrima 277 (type of the former M. fructicola 277), M. pulcherrima NRRL Y-48695 (type of the former M. andauensis), NRRL Y-48712 (type of the former M. ziziphicola) and M. pulcherrima CBS 10357 (type of the former M. sinensis) respectively.

**References**

Gardini F, Tofalo R, Belletti N, Iucci L, Suzzi G, Torriani S, Guerzoni ME, Lanciotti R (2006) Characterization of yeasts involved in the ripening of Pecorino Crotonese cheese. Food Microbiol 23(7):641-8. https://doi.org/10.1016/j.fm.2005.12.005
